# Use of Patch Augmentation in Rotator Cuff Repair: A Systematic Review Protocol

**DOI:** 10.1055/s-0045-1814406

**Published:** 2025-12-30

**Authors:** Victor Peyneau Poncio, Bianca Carolina Bankhardt da Silva, Fabio Teruo Matsunaga, Nicola Archetti Netto, João Carlos Belloti, Marcel Jun Sugawara Tamaoki

**Affiliations:** 1 Departament of Orthopedics and Traumatology, Escola Paulista de Medicina, Universidade Federal de São Paulo, São Paulo, SP, Brazil

**Keywords:** allografts, autografts, heterografts, rotator cuff, rotator cuff injuries, tissue scaffolds, alicerces teciduais, aloenxerto, autoenxertos, lesões do manguito rotador, manguito rotador, xenoenxertos

## Abstract

**Objective:**

To evaluate the effectiveness of using a graft for reinforcement in rotator cuff repair compared to no use, as well as the effectiveness of different types of grafts, considering healing and shoulder function as primary outcomes, through a systematic review and meta-analysis of randomized controlled trials (RCTs).

**Methods:**

We will conduct a systematic review of RCTs following the guidelines of the *Cochrane Handbook for Systematic Reviews of Interventions* and of the Preferred Reporting Items for Systematic reviews and Meta-Analyses for Protocols (PRISMA-P) statement. Only RCTs with adult participants comparing repair with a reinforcing graft versus the standard repair, or comparing different types of grafts, will be included. An electronic search will be conducted in the MEDLINE (PubMed), Cochrane Central Register of Controlled Trials (CENTRAL), Embase, and SciELO databases, as well as gray literature. Data management and extraction will be performed using a standardized form that will assess the characteristics of the studies, participants, and intervention, as well as the outcomes and methodological domains. The primary outcomes will be healing and shoulder functional assessment, and the secondary outcomes, pain, range of motion, adverse events, complications, quality of life, and cost-effectiveness. Risk of bias will be assessed with the Risk of Bias, version 2.0 (RoB 2.0), and the certainty of evidence, through the Grading of Recommendations Assessment, Development, and Evaluation (GRADE) approach. Descriptive, subgroup, and sensitivity analyses will be performed when applicable.

**Conclusion:**

The review is expected to consolidate evidence on the effectiveness of using a graft for reinforcement in rotator cuff repair. It has been registered in the International Prospective Register of Systematic Reviews (PROSPERO) under number 1106892.

## Resumo

**Objetivo:**

Avaliar a eficácia do uso de enxerto como reforço no reparo do manguito rotador comparado ao não uso, e a eficácia de diferentes tipos de enxerto, considerando a cicatrização e a função do ombro como desfechos primários, por meio de uma revisão sistemática e metanálise de ensaios clínicos randomizados (ECRs).

**Métodos:**

Realizaremos uma revisão sistemática de ECRs conforme as diretrizes do *Cochrane Handbook for Systematic Reviews of Interventions* e da declaração de Preferred Reporting Items for Systematic reviews and Meta-Analyses for Protocols (PRISMA-P). Serão incluídos apenas ECRs com participantes adultos que comparem o reparo com enxerto como reforço e o reparo-padrão, ou que comparem diferentes tipos de enxertos. Será realizada uma busca eletrônica nas bases MEDLINE (PubMed), Cochrane Central Register of Controlled Trials (CENTRAL), Embase e SciELO, além de literatura cinzenta. O gerenciamento e extração de dados serão conduzidos com formulário padronizado que avaliará as características do estudo, dos participantes e da intervenção, além dos resultados e dos domínios metodológicos. Os desfechos primários serão a cicatrização e a avaliação funcional do ombro, e os secundários, dor, amplitude de movimento, eventos adversos, complicações, qualidade de vida e a relação custo-eficácia. O risco de viés será avaliado por meio do Risk of Bias, versão 2.0 (RoB 2.0), e a certeza da evidência, por meio da abordagem Grading of Recommendations Assessment, Development, and Evaluation (GRADE). Quando aplicável, serão realizadas, análises descritivas, de subgrupos e de sensibilidade.

**Conclusão:**

Esta revisão espera consolidar evidências sobre a eficácia do uso de enxerto como reforço no reparo do manguito rotador. Este estudo foi inscrito no International Prospective Register of Systematic Reviews (PROSPERO) sob o número 1106892.

## Introduction

Rotator cuff injuries represent a growing challenge in shoulder surgery, as their prevalence increases with age. 1 Such injuries are the main shoulder pathology, and the surgeries to treat them are the most commonly performed in the epidemiological context of shoulder pathologies. 2 The repair failure rate, despite the constant technical advancement and scientific improvement, is significant, ranging from 30 to 40% in primary surgeries, with studies 3 4 showing predictive values of up to 80 to 90% of non-healing, depending on important factors such as healing, injury size, and the degree of atrophy of the rotator cuff muscles. This scenario directly affects patients' quality of life and the need for reinterventions, thereby increasing health costs. In the healthcare context of the United States, 5 the indirect costs of rotator cuff repair failure are estimated at hundreds of thousands of dollars.

Different strategies have been explored to minimize failures, including double-row repair, superior capsular reconstruction, and tendon transfers. 6 7 However, the placement of materials for reinforcement, called *grafts* or *patches* , has gained prominence. These materials can be biological (deriving from humans and animals) or synthetic, improving scar support and mechanical reinforcement and promoting better healing and repair resistance. 8

Recent research 9 10 suggests a 30% reduction in rupture rates and improvements in functional scores with augmentation. A survey of surgeons in the United Kigdom 11 revealed that 70% of those using augmentation do so for large or extensive injuries, but there is still disagreement over the best type of patch and the optimal indication.

Despite evidence from various studies, there is a lack of recent systematic reviews based solely on randomized controlled trials (RCTs), which justifies conducting a systematic review with meta-analysis based exclusively on RCTs. Only 3 previous reviews were found in the literature, but all were published more than 5 years ago and evaluated trials with low levels of evidence, such as non-randomized studies, case series, and retrospective studies. The overall strategy will be developed around the key concepts of the Population, Intervention, Comparison, Outcome, Study Design (PICOS) strategy: population – patients with rotator cuff injuries; intervention – augmentation with patch; comparison – repair without augmentation (control) or with another type of augmentation with graft; outcome – tendon healing and functional outcomes; and study design – systematic review.

Therefore, the objective is to compare the effectiveness of graft use versus non-use for reinforcement, as well as the different types of grafts in rotator cuff repair, considering shoulder healing and function, through a systematic review and meta-analysis of RCTs.

### Study Type

The present study is a systematic review of RCTs evaluating the effectiveness of tendon healing and functional outcomes in adults with rotator cuff injuries undergoing repair with augmentation, using any type of patch (biological or synthetic). The study will follow the recommendations proposed by the *Cochrane Handbook for Systematic Reviews of Interventions* and the Preferred Reporting Items for Systematic Reviews and Meta-Analyses for Protocols (PRISMA-P) ( Annex 1 ). 12 13

**Annex 1:** PRISMA-P 2015 checklist

Section/Topic	Item nr.	Checklist item	Page nr.
**ADMINISTRATIVE INFORMATION**			
Title			
Identification	1a	Identify the report as a protocol of a systematic review	1
Update	1b	If the protocol is for an update of a previous systematic review, identify as such	–
Registration	2	If registered, provide the name of the registry (such as PROSPERO) and registration number	1
Authors			
Contact	3a	Provide name, institutional affiliation, e-mail address of all protocol authors; provide physical mailing address of corresponding author	Title page
Contributions	3b	Describe contributions of protocol authors and identify the guarantor of the review	Title page
Amendments	4	If the protocol represents an amendment of a previously completed or published protocol, identify as such and list changes; otherwise, state plan for documenting important protocol amendments	–
Support			
Sources	5a	Indicate sources of financial or other support for the review	Title page
Sponsor	5b	Provide name for the review funder and/or sponsor	Title page
Role of sponsor or funder	5c	Describe roles of funder(s), sponsor(s), and/or institution(s), if any, in developing the protocol	Title page
**INTRODUCTION**			
Rationale	6	Describe the rationale for the review in the context of what is already known	4
Objectives	7	Provide an explicit statement of the question(s) the review will address regarding PICOS	4
**MATERIALS AND METHODS**			
Eligibility criteria	8	Specify the study characteristics (such as PICOS, setting, time frame) and report characteristics (such as years considered, language, publication status) to be used as criteria for eligibility for the review	5
Information sources	9	Describe all intended information sources (such as electronic databases, contact with study authors, trial registers or other grey literature sources) with planned dates of coverage	7
Search strategy	10	Present draft of search strategy to be used for at least one electronic database, including planned limits, such that it could be repeated	7
Study records			
Data management	11a	Describe the mechanism(s) that will be used to manage records and data throughout the review	8
Selection process	11b	State the process that will be used for selecting studies (such as two independent reviewers) through each phase of the review (that is, screening, eligibility and inclusion in meta-analysis)	8
Data collection process	11c	Describe planned method of extracting data from reports (such as piloting forms, done independently, in duplicate), any processes for obtaining and confirming data from investigators	8
Data items	12	List and define all variables for which data will be sought (such as PICOS items, funding sources), any pre-planned data assumptions and simplifications	8,9
Outcomes and prioritization	13	List and define all outcomes for which data will be sought, including prioritization of main and additional outcomes, with rationale	9
Risk of bias in individual studies	14	Describe anticipated methods for assessing risk of bias of individual studies, including whether this will be done at the outcome or study level, or both; state how this information will be used in data synthesis	10
Data synthesis	15a	Describe criteria under which study data will be quantitatively synthesised	12
	15b	If data are appropriate for quantitative synthesis, describe planned summary measures, methods of handling data and methods of combining data from studies, including any planned exploration of consistency (such as I ^2^ , Kendall's Tau)	9
	15c	Describe any proposed additional analyses (such as sensitivity or subgroup analyses, meta-regression)	11
	15d	If quantitative synthesis is not appropriate, describe the type of summary planned	10
Meta-bias(es)	16	Specify any planned assessment of meta-bias(es) (such as publication bias across studies, selective reporting within studies)	10
Confidence in cumulative evidence	17	Describe how the strength of the body of evidence will be assessed (such as GRADE)	11

**Abbreviations:** GRADE, Grading of Recommendations Assessment, Development, and Evaluation; PICOS, Population, Intervention, Comparison, Outcome, and Study Design; PRISMA-P, Preferred Reporting Items for Systematic reviews and Meta-Analyses for Protocols; PROSPERO, International Prospective Register of Systematic Reviews.

### Ethics and Records

The study was registered in the International Prospective Register of Systematic Reviews (PROSPERO) under number 1106892, with the title “Patch Augmentation in Rotator Cuff Repair: A Systematic Review and Meta-analysis of Randomized Controlled Trials.”

### Inclusion and Exclusion Criteria

Eligible articles are RCTs or quasi-randomized trials with adult participants (older than 18 years) who underwent total rotator cuff repair and compared the use of a graft as reinforcement with repair alone or with another graft.

The following exclusion criteria will be adopted: non-randomized, non-comparative, cadaveric, cohort, observational, case reports, case-control, and animal studies. Studies that include additional procedures without associated rotator cuff repair or the use of graft for another purpose (such as upper capsule reconstruction) will also be excluded.

### Primary Outcomes

The primary outcomes will be rotator cuff healing and shoulder function.

Tendon healing will be assessed using imaging methods, such as magnetic resonance imaging (MRI) or ultrasound (US). The evaluation of structural integrity will be dichotomous (presence or absence of rerupture), and it will be based on classification criteria standardized and validated in the literature, such as those proposed by Sugaya et al., 14 a classification widely used to describe postoperative structural integrity, as well as the evaluation of the absolute number of reruptures.

Shoulder function will be assessed from postoperative follow-up using validated scores such as the Constant-Murley Score (CMS), 15 the American Shoulder and Elbow Surgeons Society Standardized Shoulder Assessment Score (ASES), 16 the Penn Shoulder Score (PSS), 17 and the Disabilities of the Arm, Shoulder and Hand (DASH) questionnaire. 18 Should shoulder function be assessed using other validated scores, these will also be considered and, if applicable, their measurements will be converted to a common metric, as appropriate.

To evaluate the clinical implications of the outcomes, we will use the concept of clinically-important minimum difference (CIMD), a statistical model that defines the smallest change in a score or scale that the patient considers significant for their health status.

### Secondary Outcomes

As secondary outcomes, we will also evaluate pain (using validated scales), the range of motion, and related quality-of-life scores, such as the 36-Item Short Form Survey (SF-36). Additionally, the study will investigate and record adverse events, postoperative complications, and poor outcomes, including, but not limited to, infections, persistent stiffness, and the need for surgical reintervention.

All outcomes will be evaluated following the length of the period: short term (up to 6 months), medium term (6 months to 2 years), and long term (longer than 2 years)

### Search Methods and Strategies

The electronic search will be performed on the MEDLINE (via PubMed), Cochrane Central Register of Controlled Trials (CENTRAL), Embase, and SciELO databases. In addition, the grey literature, such as Google Scholar, will be evaluated, as well as the reference lists of the included studies and the annals of the main congresses on the subject, to search for additional relevant studies not captured in the primary electronic search. No language or publication date restrictions will be imposed.

Search strategies will be developed to maximize sensitivity and specificity. For each database, a search will be developed that combines controlled terms (when applicable, such as medical subject headings [MeSH] in PubMed/MEDLINE and Emtree in Embase) and free-text terms (keywords and synonyms), using Boolean operators (AND, OR). The overall strategy will be developed around the key concepts of the PICOS strategy ( Annex 2 ).

**Annex 2:** Search strategy

Database	Search terms	Recommended filters
MEDLINE (via PubMed)	( *Rotator Cuff* [Mesh] OR *Rotator Cuff Injuries* [Mesh] OR *rotator* cuff tear* OR rotator cuff injur* OR massive rotator cuff tear* OR large rotator cuff tear* ) AND ("Surgical Procedures, Operative"[Mesh] OR "rotator cuff repair" OR "arthroscopic repair") AND ("Biocompatible Materials"[Mesh] OR "Tissue Scaffolds"[Mesh] OR patch OR graft OR augmentation OR scaffold OR "biologic augmentation" OR "synthetic patch" OR "acellular dermal matrix") AND ("Randomized Controlled Trial"[Publication Type] OR "randomized controlled trial" OR "RCT")	Humans; Adults; Randomized clinical trials
Cochrane Central Register of Controlled Trials (CENTRAL)	(rotator cuff OR "rotator cuff tear" OR "rotator cuff injuries") AND (augmentation OR patch OR graft OR scaffold OR "acellular dermal matrix" OR "biologic augmentation" OR "synthetic patch") AND ("rotator cuff repair" OR surgery OR "arthroscopic repair")	*Trials* > *randomized*
EMBASE	('rotator cuff injury'/exp OR 'rotator cuff tear' OR 'rotator cuff repair' OR 'massive rotator cuff tear') AND ('augmentation'/exp OR 'tissue scaffold'/exp OR patch OR graft OR 'acellular dermal matrix' OR 'synthetic patch' OR 'biologic augmentation') AND ('randomized controlled trial'/exp OR 'randomized controlled trial')	Type of study: Randomized clinical trials; only humans; Age: Adult
SciELO	("manguito rotador" OR "lesão do manguito rotador" OR "rotator cuff") AND ("enxerto" OR "patch" OR "aumento" OR "augmentação" OR "scaffold" OR "matriz dérmica acelular") AND ("ensaio clínico randomizado" OR "estudo clínico randomizado" OR "randomized controlled trial")	Type of article: clinical trial

### Data Collection, Extraction, and Analysis

Two authors will independently select the studies eligible for this systematic review through the title and abstract using the following criteria: 1) RCTs; 2) presence of total rotator cuff injury; 3) surgical repair using graft reinforcement (biological, animal or human, and synthetic); and 4) comparative control group, either without augmentation or with the use of different patches. The review will be performed in two stages: primary analysis of the title and abstract, and, from the included studies, the reading of the full text. The selected studies will be reviewed in full to determine their eligibility, and any disagreement will be resolved through discussion and, when necessary, will be judged by a third party in an attempt to resolve a possible conflict. Two reviewers will perform the extraction of the data, which will be arranged in an appropriate custom form in the Excel (Microsoft Corp.) software, version 16.34, based on: 1) the characteristics of the studies, including authors, year of publication, sample size (total and per group), loss to follow-up, sociodemographic characteristics of the participants (mean age, sex, comorbidities, others), number of patients, inclusion and exclusion criteria, and follow-up time; 2) the characteristics of the intervention, such as type of surgery, use of which patch for augmentation; 3) the outcomes during the postoperative follow-up, for function (functional scores) as well as pain, range of motion, tendon healing, and complications; and 4) the methodological domains and biases.

### Data Treatment

The resulting dichotomous data will be analyzed using odds ratios (ORs) and 95%CIs. Continuous data will be expressed as mean differences (MDs) or standardized mean differences (SMDs) with 95%CIs. The outcomes will be grouped with the MD if two or more trials reveal outcomes from the same validated assessment instrument (with the same units of measurement). If the primary studies measure the same outcomes, such as shoulder function through different instruments (as well as different units of measurement), the MD will be transformed into the SMD, enabling the evaluation of the outcomes, regardless of the original units of measurement.

Statistical heterogeneity will be assessed using the Cochran's Q-test and the I ^2^ index. The Cochran's Q-test will be used to verify the statistical significance of the heterogeneity, with values of *p* < 0.10 considered indicative of significant heterogeneity, given their low power in meta-analyses with few studies. The I ^2^ index will be used to quantify the heterogeneity across studies, expressing the percentage of the total effect variation observed that is due to true heterogeneity rather than chance. The interpretation of I ^2^ will be according to the following guidelines: 0 to 40% (may not be important); 30 to 60% (moderate heterogeneity); 50 to 90% (substantial heterogeneity); and 75 to 100% (considerable heterogeneity). A random-effects model will be used to group the outcomes, given the likely clinical and methodological heterogeneity across the included studies, even when the heterogeneity test is not statistically significant. The Review Manager (RevMan, The Cochrane Collaboration) software, version 5.3, ( will be used for the statistical analyses.

When quantitative data are available for outcomes of interest, with standardizable metrics, and the heterogeneity among studies is considered acceptable, a meta-analysis will be performed. In case of significant heterogeneity, subgroup analyses and/or meta-regression will be explored, if the number of studies enables them. When applicable and with a sufficient number of studies, paired and networked meta-analyses will be considered for each outcome.

### Risk of Bias Assessment

Two authors will independently evaluate aspects of the methodological reliability of the studies using the Risk of Bias 2.0 (RoB 2, The Cochrane Collaboration) tool, 19 which is specific for RCTs, considering the four main domains addressed (bias due to deviations from the planned intervention, bias due to missing outcome data, bias in the measurement of outcomes, and bias in the selection of reported results). Disagreements between reviewers will be resolved by consensus or by a third reviewer.

### Sensitivity Analysis

The sensitivity analysis will involve the progressive exclusion of studies with heterogeneous methodological characteristics or confounding factors that may compromise the validity of the results. Studies excluded in the sensitivity analysis will not be removed from the main analysis, but will be used to assess the potential impact on the overall effect estimate. In quantitative analyses with a sufficient number of studies, a funnel graph may be used, along with Egger or Begg tests, to investigate publication bias, and the symmetry of the distribution of effects may be assessed.

### Quality of Evidence

Additionally, the quality of evidence for each critical outcome will be assessed using the Grading of Recommendations Assessment, Development and Evaluation (GRADE) approach. 20

The following criteria will be considered for the grading of evidence in the quality aspect: 1) bias due to deviations from the planned interventions; 2) bias due to missing outcome data; 3) bias in the measurement of outcomes; 4) bias in the selection of reported outcomes; 5) bias due to the randomization process; and 6) bias due to allocation concealment and blinding.

Evidence will be classified as high, moderate, low, or very low quality. Additionally, the quality of the evidence may be increased based on three factors: high magnitude of the effect, dose-response relationship, and absence of biases that explain the observed effect.

### Missing Data

An intent-to-treat analysis will be performed, including all randomized participants in each intervention. The authors of the selected trials will be contacted if insufficient information is available. An analysis will be performed regardless of data loss, according to the worst- and best-case scenarios.

### Subgroup Analysis

The subgroups will be analyzed to explore the difference in the outcomes of each graft: comparison of the use and non-use of graft in association with rotator cuff repair; comparison between the use of biological and synthetic grafts, as well as other relevant comparisons to understand the outcomes among the available techniques. In addition, the result for the subgroups will be evaluated according to the size of the injury and the number of tendons affected, if possible.

### Data Synthesis

The data synthesis will include a description of the characteristics of the included studies (e.g., population, intervention, comparison, outcomes, and risk of bias), facilitating the identification of studies eligible for meta-analysis. For the structured effects report, a random effect model and a 95% CI will be used. Consideration of the direction and magnitude of the effect, the certainty of the evidence, the interventions tested, and the populations in which they were tested will be included. The variable model will be used when there is diversity in clinical or methodological characteristics.

## Discussion

Rotator cuff injuries are one of the main shoulder pathologies. In this context, rotator cuff repair is one of the most frequently performed surgeries. One challenge that persists is the high rate of repair failure, whether due to re-healing or non-healing of the tendon. This has important implications for the patient's quality of life and the need for reinterventions, and it is essential to evaluate the effectiveness of different treatment strategies. The use of graft reinforcement is an explored technique that acts as mechanical and biological reinforcement for repair.

A systematic review and meta-analysis of RCTs constitutes high-level scientific evidence for assessing the effectiveness of treatments. As primary outcomes, we will use objective measures of tendon healing via MRI or US and functional improvement, as assessed with validated scores. This combination provides an overview of the clinical and structural success of the procedure. The evaluation across short-, medium-, and long-term periods provides evidence on the longevity of the results. Additionally, we will investigate secondary outcomes, including pain, range of motion, complications, and adverse events. The use of subgroup analyses and the evaluation of heterogeneity allow us to observe differences in outcomes across different graft types and injury sizes, as the magnitude of the benefit can be influenced by graft material, the technique employed, and patient characteristics, reinforcing the need for recommendations.

Considering that the current literature addresses several treatment options for rotator cuff injuries, there is still a lack of high-level scientific evidence to support clinical practice. Therefore, this systematic review will consolidate existing evidence and clarify the role of this technique in rotator cuff repair.
